# How HIV-1 Integrase Associates with Human Mitochondrial Lysyl-tRNA Synthetase

**DOI:** 10.3390/v12101202

**Published:** 2020-10-21

**Authors:** Xaysongkhame Phongsavanh, Noha Al-Qatabi, Mohammed Samer Shaban, Fawzi Khoder-Agha, Merwan El Asri, Martine Comisso, Raphaël Guérois, Marc Mirande

**Affiliations:** Institute for Integrative Biology of the Cell (I2BC), French Alternative Energies and Atomic Energy Commission (CEA), French National Centre for Scientific Research (CNRS), Université Paris-Sud, Université Paris-Saclay, 1 Avenue de la Terrasse, 91190 Gif-sur-Yvette, France; phongsavanh.micky@hotmail.com (X.P.); alqatabinoha@gmail.com (N.A.-Q.); mohammed.s.shaban@pharma.med.uni-giessen.de (M.S.S.); fawzi.khoderagha@outlook.com (F.K.-A.); elasrimerwan@hotmail.fr (M.E.A.); martine.comisso@i2bc.paris-saclay.fr (M.C.); raphael.guerois@cea.fr (R.G.)

**Keywords:** tRNA packaging complex, HIV-1, mitochondrial lysyl-tRNA synthetase, integrase, 3D model

## Abstract

Replication of human immunodeficiency virus type 1 (HIV-1) requires the packaging of tRNA^Lys,3^ from the host cell into the new viral particles. The GagPol viral polyprotein precursor associates with mitochondrial lysyl-tRNA synthetase (mLysRS) in a complex with tRNA^Lys^, an essential step to initiate reverse transcription in the virions. The C-terminal integrase moiety of GagPol is essential for its association with mLysRS. We show that integrases from HIV-1 and HIV-2 bind mLysRS with the same efficiency. In this work, we have undertaken to probe the three-dimensional (3D) architecture of the complex of integrase with mLysRS. We first established that the C-terminal domain (CTD) of integrase is the major interacting domain with mLysRS. Using the *p*Bpa-photo crosslinking approach, inter-protein cross-links were observed involving amino acid residues located at the surface of the catalytic domain of mLysRS and of the CTD of integrase. In parallel, using molecular docking simulation, a single structural model of complex was found to outscore other alternative conformations. Consistent with crosslinking experiments, this structural model was further probed experimentally. Five compensatory mutations in the two partners were successfully designed which supports the validity of the model. The complex highlights that binding of integrase could stabilize the tRNA^Lys^:mLysRS interaction.

## 1. Introduction

The human immunodeficiency virus type 1 (HIV-1) is a retrovirus that contains two copies of its genomic single-stranded RNA embedded into the nucleocapsid of mature particles. During the assembly of the virus, some tRNAs from the host cell are selectively packaged into the budding particles [1,2]. This includes tRNA^Lys,3^ that serves as a primer for initiation of reverse transcription, an essential step of the life cycle of the virus that is believed to take place in the virus shortly after budding, before infection of host cells [3]. In virio analyses reveal that annealing of tRNA^Lys,3^ to the primer binding site (PBS) of viral RNA occurs in the viruses [4], consistent with the finding that the level of viral infectivity is correlated with the level of tRNA^Lys,3^ encapsidation into the virions [5]. Lysyl-tRNA synthetase, an enzyme of the translation machinery that catalyzes aminoacylation of tRNA^Lys,3^ with lysine, is also detected in HIV-1 virions [6,7] and its interactions with Gag and GagPol polyproteins is suggested to be essential for tRNA^Lys,3^ encapsidation into newly formed viral particles [8,9]. The early model suggested that cytoplasmic LysRS, after dissociation from the multisynthetase complex, following phosphorylation on Ser207 by MAPK, associates with Gag [6,10]. Monospecific antibodies directed to human mitochondrial lysyl-tRNA synthetase (mLysRS) identified a cross-reactive protein in extracts of HIV-1 particles [7], corresponding to the mature form of mLysRS produced after cleavage of its N-terminal mitochondria-targeting sequence [11]. The tRNA^Lys,3^ packaging complex is formed by the association of GagPol with the LysRS:tRNA^Lys,3^ complex. The catalytic domain of mLysRS was shown to interact with the transframe and integrase domains of the GagPol polyprotein precursor [8]. The interaction between mLysRS and the integrase subunit (IN) from the Pol domain of the GagPol precursor is the major contributor to the stability of the GagPol:mLysRS complex [12].

In human, cytoplasmic and mitochondrial LysRSs are encoded by the *KARS1* gene by means of alternative splicing [13]. They share 576 amino acid residues in common. The cytoplasmic and the mature mitochondrial enzymes possess specific N-terminal sequences of 21 and 19 amino acid residues, respectively [11]. They are homodimers composed of a C-terminal catalytic domain, a central tRNA anticodon-binding domain, and a eukaryote-specific N-terminal domain that stabilizes the LysRS:tRNA^Lys^ complex [11,14]. In the crystal-structure of LysRS, only the catalytic and tRNA anticodon-binding domains are visible [15]. These two domains are shared by the cytoplasmic and mitochondrial LysRS.

After maturation of the GagPol precursor by viral protease, the integrase (IN) from HIV-1 is released as a dimer formed of two identical subunits made of 288 amino acid residues, which also forms a dimer of dimers at high concentration, or when it binds RNA [16,17]. The primary function of integrase is integration of viral DNA into the host genome. Integrase strand-transfer inhibitors (INSTIs) that target this function have been developed and are used to treat HIV-1 infections [18]. Recent studies showed that IN is also essential during morphogenesis of the particles [17]. It binds viral RNA and is necessary for proper localization of the viral genome inside the capsid. Allosteric IN inhibitors (ALLINIs) define a new class of antiretroviral agents that target IN oligomerization and compromise viral replication [19]. HIV-1 integrase is constituted of three distinct structural domains, a α-helical N-terminal domain (IN-NTD), the catalytic core domain (IN-CCD), and a β-barrel C-terminal domain (IN-CTD). Crystal structures of IN-CCD alone, or of IN-CCD with either the NTD or the CTD have been reported [20,21]. The structural domains are linked by long spacer polypeptides which are not always visible in the crystal structures. The three domains of integrase are clearly visible in the cryo-EM structure of the HIV-1 strand transfer complex intasome [22]. Interactions of integrase with viral and target DNA within the intasome involve the CCD and CTD of integrase, and the NTD-CCD linker.

The knowledge of the three-dimensional (3D) structure of the three structural domains of integrase and of the catalytic and anticodon-binding domains of LysRS opens the way to explore the mechanism of mLysRS:IN association. In this work, we conducted a detailed analysis of the complex of mLysRS with the integrase from HIV-1. We first determined that the CTD of IN from HIV-1 is responsible for its association with mLysRS. HIV-2 is a retrovirus that also uses tRNA^Lys,3^ as a primer for reverse transcription but displays significant sequence variations in its genome, including the integrase coding sequence. We determined that IN from HIV-2 binds mLysRS with an efficiency similar to IN from HIV-1. Then, we developed experimental and computational approaches in order to propose a structural model of the mLysRS:IN complex. Finally, this structural model was subjected to mutational probing. In particular, five compensatory mutants were constructed on the basis of the structural model, which eventually led to the validation of a 3D model of mLysRS:IN interaction. The combination of experimental and in silico approaches used in this study allowed to determine the mode of interaction of mLysRS with IN, an interaction believed to be essential for the replication of the virus. Targeting the mLysRS:IN-CTD complex with inhibitors of its assembly may prove useful to develop new antiviral drugs with original resistance profiles.

## 2. Materials and Methods

### 2.1. Expression of HIV-1 Integrase in E. coli and Purification

The HIV-1 integrase coding region from pNL4-3 (nucleotides 4230 to 5093) was amplified by PCR with oligonucleotides GP119 (5′-GTTTAACTTTAAGAAGGAGATATACCATGGCGTTTTTAG ATGGAATAGATAA) and GP159 (5′-ATCTCAGTGGTGGTGGTGGTGGTGCTCGAGATCCTCATC CTGTCTACTTG), and introduced by the SLIC method [23] into pET28b digested with NcoI and XhoI. For cloning of IN-∆N, PCR was performed with primers GP120 (5′-GTTTAACTTT AAGAAGGAGATATACCATGGCGATGCATGGACAAGTAGACTG) and GP159, and for cloning IN-CCD, primers were GP120 and GP160 (5′-ATCTCAGTGGTGGTGGTGGTGGTGCTCGAGTTCTT TAGTTTGTATGTCTGTTG). For cloning of IN-CTD213 and IN-CTD222, the 5′-primers were IN003 (5′-GTTTAACTTTAAGAAGGAGATATACCATGGCGTTACAAAAACAAATTACAAAAATTC) and IN005 (5′-GTTTAACTTTAAGAAGGAGATATACCATGGCGAATTTTCGGGTTTATTACA GG), respectively, and the 3′-primer was GP159. For cloning of IN-CTD222∆C5, IN-CTD222∆C10 or IN-CTD222∆C15, the 5′-primer was IN005, and the 3′-primers were IN104 (5′-ATCTCAGTGGTGGTGGTGGTGGTGCTCGAGACTTGCCACACAATCATCACCTGC), IN105 (5′-ATCTCAGTGGTGGTGGTGGTGGTGCTCGAGATCACCTGCCATCTGTTTTCCA), or IN106 (5′-ATCTCAGTGGTGGTGGTGGTGGTGCTCGAGTTTTCCATAATCCCTGATGATCT), respectively. The sequence of the recombinant plasmids was verified by DNA sequencing. A C-terminal His-Tag is appended to all these constructs.

The proteins (IN-H^6^, IN-∆N-H^6^, IN-CCD-H^6^) were expressed in *E. coli* BL21(DE3) grown in LB medium supplemented with kanamycin (50 µg/mL). Cultures (6 liters) were grown at 37 °C to an *A*_600_ = 0.20, transferred to 15 °C, and grown to an *A*_600_ = 0.5, and expression was induced by addition of 1 mM IPTG for 16 h. Cells were collected by centrifugation (3000× *g*, 10 min, 4 °C), washed twice with ice-cold buffer 150/10 (20 mM K-phosphate pH 7.5, 150 mM NaCl, 10 mM imidazole, 5% glycerol, 5 mM 2-mercaptoethanol), resuspended in the same buffer (1 mL per g of cell pellet) and lysed in an Eaton Press after freezing in dry ice. All subsequent steps were conducted at 4 °C. After addition of 2 vol. of buffer 150/10 supplemented with protease inhibitors (1 mM Pefabloc, 10 mM benzamidine, and 10 mM PMSF), extracts were clarified by sonication and by centrifugation at 70,000× *g* for 30 min, and incubated for 1 h at 4 °C with 1 mL of Ni-NTA Superflow matrix (Qiagen, Hilden, Germany). Beads were extensively washed with buffer 500/50 (20 mM K-phosphate pH 7.5, 500 mM NaCl, 50 mM imidazole, 5% glycerol, 5 mM 2-mercaptoethanol), and elution was performed in Bio-Spin columns (BioRad, Hercules, CA, USA) by adding 6 × 1 mLof buffer 500/400 (20 mM K-phosphate pH 7.5, 500 mM NaCl, 400 mM imidazole, 5% glycerol, 5 mM 2-mercaptoethanol) and centrifugation 1 min at 1500× *g*.

Eluted proteins were either dialyzed against buffer ASU (20 mM Tris-HCl pH 7.5, 100 mM NaCl, 500 mM Urea, 10% glycerol, 1 mM EDTA, 10 mM 2-mercaptoethanol) for IN-H^6^ and IN-∆N-H^6^ (pI = 8.74 and 9.46, respectively), or against buffer AQU (20 mM Tris-HCl pH 7.5, 500 mM Urea, 10% glycerol, 1 mM EDTA, 10 mM 2-mercaptoethanol) for IN-CCD-H^6^ (pI = 7.8), and applied either to a Mono S HR 5/5 column (IN-H^6^ and IN-∆N-H^6^) or to a Mono Q HR 5/5 column (IN-CCD-H^6^) equilibrated in the same buffers. Proteins were eluted by linear gradients (40 column vol.) of NaCl from 100 to 300 mM (Mono S) or from 0 to 300 mM (Mono Q). Purified proteins were concentrated by ultrafiltration (Vivaspin 6, 10 kDa), dialyzed against storage buffer (20 mM K-phosphate pH 7.5, 100 mM NaCl, 0.02% Triton X-100, 2 mM DTT), and stored at −80 °C. Protein concentrations were determined by using calculated absorption coefficients of 1.578, 1.632, or 1.552 *A*_280_ units mg^−1^ cm^2^, respectively for IN-H^6^, IN-∆N-H^6^, or IN-CCD-H^6^.

The various derivatives of the CTD of IN (IN-CTD213-H^6^, IN-CTD222-H^6^, IN-CTD222∆C5-H^6^, IN-CTD222∆C10-H^6^, and IN-CTD222∆C15-H^6^) were expressed as described above, except that cells were grown at 37 °C to an *A*_600_ = 0.25, transferred to 28 °C and grown to an *A*_600_ = 0.5, and expression was induced by addition of 1 mM IPTG for 6 h. Cell lysis and Ni-NTA chromatography were conducted as described above. Purification on Mono S HR 5/5 columns was performed as described above, except that elution was achieved by gradients (50 column vol.) of NaCl from 100 to 500 mM. Purified proteins were concentrated by ultrafiltration (Vivaspin 6, 3 kDa), dialyzed against storage buffer (20 mM K-phosphate pH 7.5, 100 mM NaCl, 2 mM DTT), and stored at −80 °C. Protein concentrations were determined by using calculated absorption coefficients of 1.536, 1.72, 1.853, 1.965, or 2.099 *A*_280_ units mg^−1^ cm^2^, respectively for IN-CTD213-H^6^, IN-CTD222-H^6^, IN-CTD222∆C5-H^6^, IN-CTD222∆C10-H^6^, and IN-CTD222∆C15-H^6^.

### 2.2. Expression of HIV-2 Integrase in E. coli and Purification

The coding regions of integrase from the two strains HIV-2_ALL and HIV-2_TRA, were amplified by PCR with oligonucleotides IN001 (5′-GTTTAACTTTAAGAAGGAGATATACCATGGC GTTTTTAGAGAACATAGAACC) and IN101 (5′-ATCTCAGTGGTGGTGGTGGTGGTGCTCGAGT GCTTCAGGTACTTGACCAGAC) or IN002 (5′-GTTTAACTTTAAGAAGGAGATATACCATGGCG TTCCTAGAAAAAATAGAACC) and IN102 (5′-ATCTCAGTGGTGGTGGTGGTGGTGCTCGAGC CTGGTATCCTCCACGTCGGCA), respectively, and introduced by the SLIC method into pET28b digested with NcoI and XhoI.

The two integrase species from HIV-2_ALL and HIV-2_TRA were expressed in *E. coli* BL21(DE3) grown at 37 °C to an *A*_600_ = 0.25, transferred to 28 °C and grown to an *A*_600_ = 0.5, and expression was induced by addition of 1 mM IPTG for 6 h. Purification was conducted exactly as described above for IN-H^6^ from HIV-1. Protein concentrations were determined by using calculated absorption coefficients of 1.156 and 1.203 *A*_280_ units mg^−1^ cm^2^, respectively, for IN from HIV-2_ALL and HIV-2_TRA.

### 2.3. HTRF Assay

Homogeneous time-resolved fluorescence (HTRF) assays were performed in black, flat bottom, half-area, non-binding surface, 96-well microplates (Corning #3993). Human mitochondrial LysRS (mLysRS) with a C-terminal HA-tag (mLysRS-HA) was incubated at a dimer concentration of 1.5 nM with the various integrase derivatives carrying a C-terminal His-tag at concentrations indicated in the legends of the figures, in 10 mM Tris-HCl pH 7.5, 50 mM NaCl, 10 mM 2-mercaptoethanol, and BSA at 1 mg/mL, for 1 h on ice. Antibodies (Cisbio) directed to the His-tag and conjugated with Eu^3+^ cryptate (Cisbio #61HISKLB), and to the HA-tag conjugated with XL665 (Cisbio #610HAXLB), were added and incubation was continued for 30 min. After addition of 50 mM KF, fluorescence of Eu^3+^ cryptate and of XL665 was recorded at 620 nm (*I*_620_) and 665 nm (*I*_665_), respectively, after excitation of Eu^3+^ cryptate at 317 nm, in an Infinite M1000 PRO microplate reader (TECAN). Results are expressed as the ratio of *I*_665_/*I*_620_.

### 2.4. Protein Photo-Cross-Linking

The QuickChange Lightning Site-directed Mutagenesis Kit from Agilent Technologies was used to introduce amber (TAG) stop codons at discrete sites within the nucleotide sequence encoding the catalytic domain of mLysRS or the CTD222 domain of integrase from HIV-1 as described previously [24]. Incorporation of *p*-benzoyl-L-phenylalanine (*p*Bpa) into mutant proteins was conducted in *E. coli* BL21(DE3) transformed with pEVOL-Bpa that expresses the orthologous supression system [25]. The *E. coli* strains containing pET28b expressing the various mLysRS or IN-CTD222 mutants carrying TAG stop codons were grown at 37 °C in 1L of LB medium supplemented with kanamycin (50 µg/mL) and chloramphenicol (34 µg/mL), and containing 0.2% arabinose and 1 mM *p*Bpa (Bachem, Bubendorf, Switzerland). When the culture reached an *A*_600_ = 1.0, expression was induced by addition of 1 mM IPTG for 5 h. The mLysRS*^p^*^Bpa^ variants with *p*Bpa inserted at the 35 distinct positions listed in Table S2 were purified as described previously [24], and the IN-CTD222*^p^*^Bpa^ variants with *p*Bpa inserted at the 12 distinct positions listed in Table S3 were purified as described above for wild-type IN-CTD222-H^6^. All the proteins were dialyzed against PBS and stored at −80 °C. During their purification, all the mutants of mLysRS*^p^*^Bpa^ or IN-CTD222*^p^*^Bpa^ were eluted similarly to their wild-type counterparts, mLysRS^WT^ or IN-CTD222^WT^, suggesting that their oligomeric structure was not affected by insertion of *p*Bpa, as expected for mutations of residues accessible to the solvent.

Photo-cross-linking was conducted essentially as described in [26]. The different mLysRS*^p^*^Bpa^ species and IN-CTD222*^p^*^Bpa^ species were mixed with IN-CTD222^WT^ and mLysRS^WT^, respectively, in a final volume of 80 µL into the wells of a 96-well plate cooled on ice, at protein concentrations indicated in the legends of the figures. Plates were covered with their polystyrene lids and with 3 mm-thick glass plates to filter short-wavelength UV light, and incubated on ice into a CL-1000 Ultraviolet Crosslinker (UVP) equipped with a 365 nm UV lamp. Control samples were withdrawn before starting irradiation, and cross-linked products were analyzed by SDS-PAGE and western blotting after exposure to UV light.

### 2.5. Docking Simulation for the mLysRS and IN Domains

Rigid-body docking was performed between a dimeric structure of lysine-tRNA synthetase and a crystal structure of the C-terminal CTD domain of HIV-1 integrase. The input structure used for lysyl-tRNA synthetase (PDB: 3BJU) was crystallized without a tRNA molecule. The catalytic and tRNA anticodon-binding domains visible in the crystal structure are shared by cytoplasmic and mitochondrial LysRS. We modeled the structure of the bound tRNA using as template the yeast tRNA:aspartyl-tRNA synthetase complex structure (PDB: 1ASY) and superimposed the synthetase chains to deduce the conformation of the bound tRNA. An input structure containing the bound tRNA was preferred so as to prevent docking models to accumulate in unlikely regions where tRNA binds. As for the HIV-1 integrase partner, the input structure was a homodimeric structure containing both the catalytic core and the CTD (PDB: 1EX4). For the docking step, we did not isolate the CTD domain of the HIV-1 integrase, and rather kept all the domains present in the 1EX4 homodimeric structure. In that way, the helical linker connecting the catalytic core and the CTD prevented docking models to accumulate in unlikely regions.

The rigid-body docking step was performed using the InterEvDock2 server [27] that takes into account both the physicochemical nature of protein surfaces and co-evolutionary information and uses three complementary scores Frodock [28], SOAP-PP [29] and InterEvScore [30] to identify the most likely interfaces (http://bioserv.rpbs.univ-paris-diderot.fr/services/InterEvDock2/). In the context of this particular complex, no co-alignments between both partners could be generated since they belong to different species. The docking protocol was performed as described previously following the standard protocol of the server [31,32], using as input the two dimeric structures described above. In the result archive, the 50 best decoys of every three scores (Frodock, SOAP-PP, InterEvScore) used in the consensus selection of the docking models were considered. Among those 150 models, 119 solutions involved the IN-CTD in the interface with LysRS. For the next refinement steps, only the CTD and not the catalytic core domain was considered in the structural models of the complex with LysRS. Models were clustered using fcc [33] with a cutoff threshold of 0.5 and removing similarities between symmetrical structures. Forty-nine non-redundant representative models of complexes were retrieved and were refined using Rosetta [34] through a standard relax protocol under native coordinate constraints and the scoring of the resulting interface energy between IN-CTD and LysRS using the beta_nov15 scoring function. The model with the lowest interface energy reached −45.9 rosetta units, significantly lower than any of the alternative configurations (second best model at −41.3) and was first selected for in-depth structural analysis and design of disruptive compensatory mutants. The coordinates of the refined structural model were deposited on the ModelArchive database (DOI: 10.1016/j.str.2008.12.014) and can be downloaded at (https://modelarchive.org/doi/10.5452/ma-bxirn).

### 2.6. Yeast Two-Hybrid Analysis

The yeast two-hybrid system developed by Brent et al. was used [35]. The mLysRS and IN-CTD222∆C10 coding sequences were inserted into the plasmids pJG4-5 (fused to the B42-activator domain placed under the control of a galactose-inducible promoter) and pEG202 (fused to the LexA DNA binding domain), respectively. Mutations in mLysRS or IN-CTD222∆C10 coding sequences listed in Table S3 were generated using the QuickChange Lightning Site-directed Mutagenesis Kit (Agilent Technologies, Santa Clara, CA, USA). The yeast strain SKY54 (*Mat*α *his3 leu2*::*3LexAop-LEU2 ura3 trp1 lys2::λcI-op-LYS2*) [36], which contains a chromosomal *LEU2* gene placed under the control of LexA operators was transformed to his^+^ with pEG202-derivatives and to trp^+^ with pJG4-5 derivatives. At least four independent colonies were analyzed for their ability to grow in the absence of leucine (expression of *LexAop-LEU2*). SKY54 expressing a pair of interactive proteins grew on galactose medium (YNBGal) lacking leucine (expression of B42-fusions that interacted with LexA-fusions) but did not grow on glucose medium (YNB) lacking leucine (no expression of B42-fusions).

### 2.7. Antibodies and Western Blot Analysis

Rabbit anti-IN-CTD antibodies were generated against a synthetic peptide (NFRVYYRDSRDPV WKGPAKLLWKGEGAVVIQDNSDIKVVPRRKAKIIRDYGK) corresponding to residues 222-273 of HIV-1 integrase and affinity purified (GeneCust, Boynes, France). The specificity of these antibodies was controlled by western blotting (Figure S1). Polyclonal antibodies to LysRS have been described previously [37]. Western blot analyses were conducted with goat anti-rabbit secondary antibodies conjugated to peroxidase (Chemicon) and the SuperSignal West Pico chemiluminescent substrates (Thermo Scientific, Waltham, MA, USA). Chemiluminescence was detected with a LAS-3000 Imaging System (Fuji, Tokyo, Japan).

## 3. Results

### 3.1. The C-Terminal Domain Is the Major Region of Integrase Interacting with mLysRS

**Figure 1:**
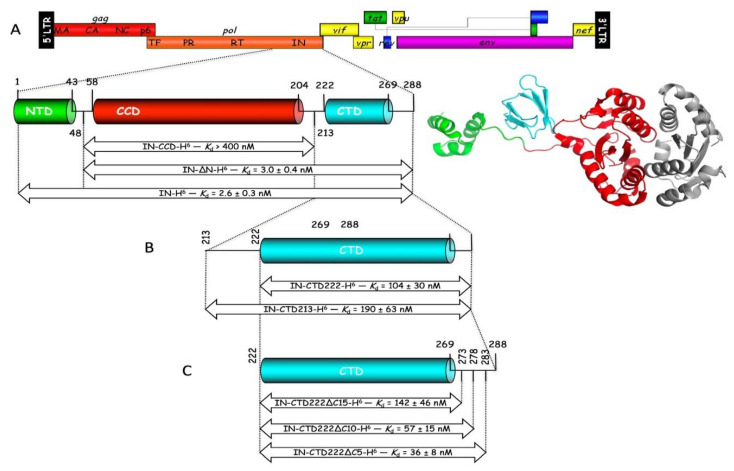
The three-domain structure of integrase from HIV-1, and the constructs of IN used in this study. (**A**): The complete genome of HIV-1 is shown. Integrase (IN) is encoded in the very C-terminal region of the Pol domain from the GagPol polyprotein precursor. The schematic view and three-dimensional (3D) representation of IN are shown. The domain structure of IN is formed by the N-terminal domain (NTD, in green) and the C-terminal domain (CTD, in cyan) connected to the catalytic core domain (CCD, in red). The second CCD of the IN dimer visible in the cryo-EM structure is shown in grey [22]. Full-length integrase with a C-terminal His-tag (IN-H^6^), with a deletion of the NTD (IN-∆N), or with a deletion of the NTD and the CTD (IN-CCD) were constructed. (**B**) Constructs of the CTD on IN starting at residues 213 (IN-CTD213) or 222 (IN-CTD222) are shown. (**C**) Constructs of IN-CTD222 with a deletion of 5, 10, or 15 C-terminal residues. These derivatives of IN were tested for their association with mLysRS (*K*_d_ determined as reported in Figure 2 are indicated; values are the means ± SEM of three independent experiments).

The packaging of tRNA_3_^Lys^ from the host cell within newly made HIV-1 particles involves the formation of a ternary complex comprising the GagPol viral polyprotein, mitochondrial lysyl-tRNA synthetase and tRNA^Lys^ from the host cell. The integrase domain located at the very C-terminus of the GagPol precursor protein (Figure 1) is the main contributor to the stability of the complex between mLysRS and GagPol [12]. The cryo-EM structural analysis of the HIV-1 STC intasome revealed that integrase is made of three well-defined structural domains (Figure 1). The α-helical N-terminal domain (NTD) and the β-barrel C-terminal domain (CTD), are connected to the catalytic core domain (CCD) via long spacer polypeptides of 15 and 18 amino acid residues, respectively [22].

**Figure 2:**
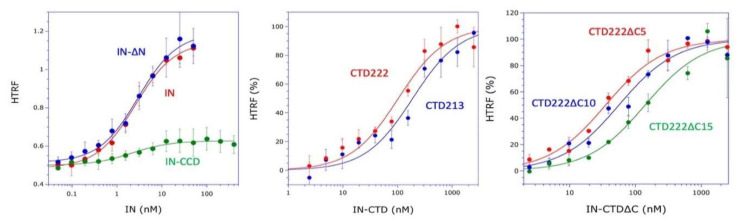
Association of the derivatives of IN with mLysRS. The binding affinities of mLysRS to IN constructs were determined in an HTRF assay using 1.5 nM of HA-tagged mLysRS and increasing concentrations of His-tagged IN derivatives, expressed as dimer (IN, IN-∆N, IN-CCD) or monomer (CTD213, CTD222, CTD222∆C5, CTD222∆C10, CTD222∆C15) concentrations. Experimental values (symbols) were fit (curves) to a binding equation assuming that one molecule of IN binds one molecule of mLysRS. The binding constants and the associated standard deviations (*n* = 3) are indicated in Figure 1.

To determine which domain of HIV-1 integrase interacts with mLysRS, integrase was expressed in *E. coli* with a C-terminal His-tag (IN-H^6^), as well as two derivatives with a deletion of the NTD (IN-∆N-H^6^) or of the NTD and the CTD (IN-CCD-H^6^) (Figure 1A). These three constructs contain the CCD dimerization domain of IN. The apparent dissociation constants of the complexes formed between these three integrase species and mLysRS were determined using a homogeneous time-resolved fluorescence (HTRF) assay described in Khoder-Agha et al. [12]. Whereas the binding affinity determined for IN-∆N-H^6^ (*K*_d_ of 3.0 ± 0.4 nM) was similar to that determined for native integrase (IN-H^6^; *K*_d_ of 2.6 ± 0.3 nM), no interaction could be detected with CCD-H^6^ (*K*_d_ > 400 nM) (Figure 2). Thus, association of IN-∆N-H^6^ with mLysRS was lost upon removal of the CTD.

These data suggested that the CTD of IN is the major interacting domain with mLysRS. Two forms of the CTD of IN were expressed in *E. coli* (Figure 1B). The IN-CTD213-H^6^ species exactly corresponds to the domain removed from IN-∆N-H^6^ to give CCD-H^6^ described above. In addition, the IN-CTD222-H^6^ derivative was obtained. It corresponds to the complete removal of the spacer located between the CCD and the CTD domains of IN. These two constructs are supposed to be monomeric proteins at the concentrations used in this study. The two IN-CTD derivatives interacted with mLysRS with binding affinities of 190 ± 63 nM and 104 ± 30 nM, respectively, for IN-CTD213-H^6^ and IN-CTD222-H^6^ (Figure 2). The loss of affinity observed between IN and the CTDs is consistent with the fact that IN is an oligomeric protein containing several CTDs, which provides a synergy of interaction with the two binding domains of a dimer of mLysRS. 

The two IN-CTD213-H^6^ and IN-CTD222-H^6^ constructs possess a 19-amino acid long C-terminal flanking polypeptide not visible neither in the crystal structure of IN-CCD-CTD (1EX4), nor in the NMR structure of IN-CTD (1IHV), which suggests that this peptide is disordered. Three derivatives of IN-CTD222-H^6^ were obtained, with a deletion of five (IN-CTD222∆C5-H^6^), 10 (IN-CTD222∆C10-H^6^), or 15 (IN-CTD222∆C15-H^6^) C-terminal residues (Figure 1C). These three CTD derivatives interacted with mLysRS with binding affinities of 36 ± 8 nM, 57 ± 15 nM, and 142 ± 46 nM (Figure 2), respectively, showing that the β-barrel domain (Figure 1A), from residues 222 to 269, is the main region of IN-CTD involved in the interaction with mLysRS.

### 3.2. Integrase from HIV-2 Also Interacts with mLysRS 

**Figure 3:**
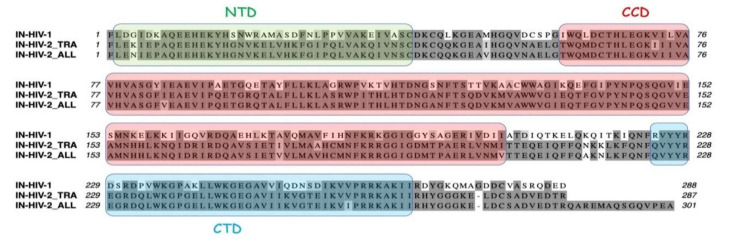
Sequence alignment of integrases from HIV-1 and HIV-2. Integrase from HIV-1 of pNL4-3 (IN_HIV-1) and two integrase species from two HIV-2 isolates (IN-HIV-2_TRA and IN-HIV-2_ALL) are aligned. Residues conserved in at least two sequences are outlined in dark grey, similar residues are in light grey. The three structural domains (NTD, CCD, and CTD) are indicated according to Figure 1A.

Integrase from HIV-2 displays only 60% of identical residues as compared to integrase from HIV-1 (Figure 3 and Table S1). The more variable domain is the NTD, with only 53.3% of identical residues between HIV-1 and HIV-2, the CCDs are more conserved, with 64.1% of identical residues between HIV-1 and HIV-2, and the highest level of conservation is observed for the CTDs, with about 70% of identities between HIV-1 and HIV-2.

**Figure 4:**
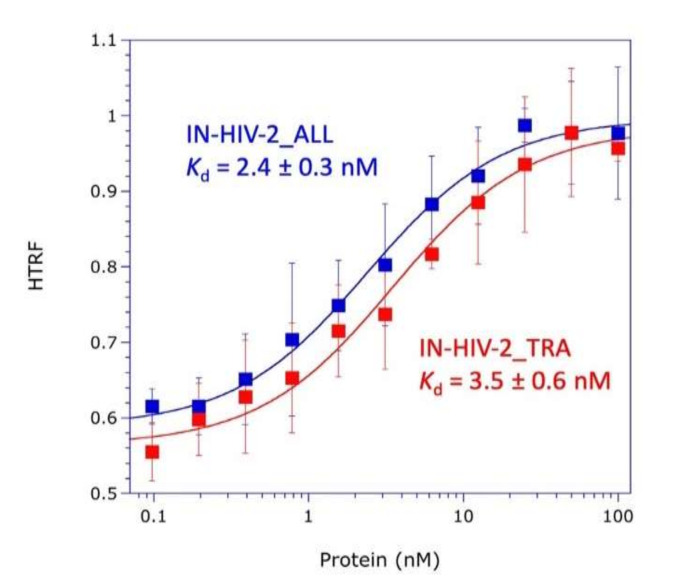
Association of IN from HIV-2 with mLysRS. The binding affinities of mLysRS to IN-HIV-2_TRA and IN-HIV-2_ALL were determined in an HTRF assay using 1.5 nM of HA-tagged mLysRS and increasing concentrations of His-tagged IN-HIV-2 species, expressed as dimer concentrations. Experimental values (symbols) were fit (curves) to a binding equation assuming that one molecule of IN binds one molecule of mLysRS. The binding constants and the associated standard deviations (*n* = 3) are indicated.

Because HIV-2 also uses the host tRNA_3_^Lys^ as a primer for initiation of the reverse transcription process, it is probable that HIV-2 also uses mLysRS to form the tRNA_3_^Lys^ packaging complex. The CTD of integrase from HIV-1 is responsible for the interaction with mLysRS (Figure 1), and it is noticeable that the CTD domains of integrases from HIV-1 and HIV-2 are the most conserved regions. The two forms of integrase from two HIV-2 isolates were expressed with a C-terminal His-tag, IN from HIV-2_TRA and from HIV-2_ALL. The two integrases from HIV-2 interacted with mLysRS with binding affinities of 3.5 ± 0.6 nM and 2.4 ± 0.3 nM, respectively, for IN from HIV-2_TRA and IN from HIV-2_ALL, which is similar to the value of 2.6 ± 0.3 nM observed for IN of HIV-1 (Figure 4). These data indicate that the binding site of IN for mLysRS are conserved between HIV-1 and HIV-2.

### 3.3. Identification of the Amino Acid Residues of mLysRS Involved in Its Interaction with the CTD of Integrase

**Figure 5:**
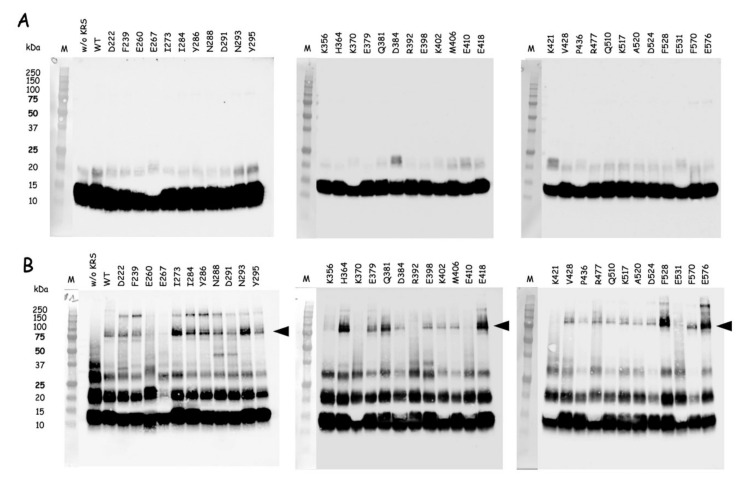
Cross-linking of the *p*Bpa-containing mLysRS variants with IN-CTD222. Wild-type mLysRS (WT) or mLysRS containing *p*Bpa inserted at different positions (D222 to E576) were incubated at a dimer concentration of 85 nM with IN-CTD222 (monomer concentration of 1.12 µM) and exposed to UV light at 365 nm for 70 min. Samples recovered before (**A**) or after (**B**) exposure to UV were analyzed by SDS-PAGE and western blotting using anti-IN-CTD antibodies. The lanes containing the stained size-marker are added on the left (in kDa). The migration level corresponding to an expected cross-linked polypeptide (79 kDa) containing one monomer of IN-CTD222 and one monomer of mLysRS is indicated by a black arrowhead. This experiment is the representative of two independent experiments.

To identify the surface area of mLysRS involved in its interaction with the CTD of integrase from HIV-1, 35 variants of mLysRS carrying each a single *p*Bpa inserted at the surface of the protein and evenly distributed at the surface of the catalytic domain (Table S2) were isolated as described previously [24]. The wild-type and *p*Bpa-containing mLysRS proteins were incubated on ice at a dimer concentration of 85 nM in the presence of IN-CTD222-H^6^ at a monomer concentration of 1.12 µM, in PBS buffer. After 70 min of exposure to UV at 365 nm, samples were analyzed by SDS-PAGE and western blotting using anti-IN-CTD antibodies (Figure 5). After incubation with seven of the *p*Bpa-containing mLysRS mutants, a high-intensity polypeptide with a molecular mass of 79 kDa was observed, corresponding to the expected size for a cross-linked species containing one monomer of IN-CTD222-H^6^ per monomer of mLysRS. Four of the mutants, with *p*Bpa inserted at positions I273, N293, F528, and E576, formed a patch of residues located in the vicinity of each other (Figure S2). This patch is formed by three residues from one monomer (I273, N293, F528) and one residue from the other monomer (E576). The three other *p*Bpa-containing proteins corresponded to insertion of *p*Bpa at positions H364, Q381, and E418, which are scattered at the surface of mLysRS. Among the other mutants of mLysRS, insertion of *p*Bpa at positions I284 and Y286, residues located within the patch of residues, defined above, also yielded a significant level of cross-linking. The identified residues define one site of interaction located on one side of the dimer of mLysRS, between the acceptor arms of the two tRNA molecules (Figure S2). Because mLysRS is a symmetrical dimer, the two equivalent patches that are located 30 Å apart could bind the two CTDs of the native dimeric integrase.

### 3.4. Identification of the Amino Acid Residues of the CTD of Integrase Involved in Its Interaction with mLysRS

**Figure 6:**
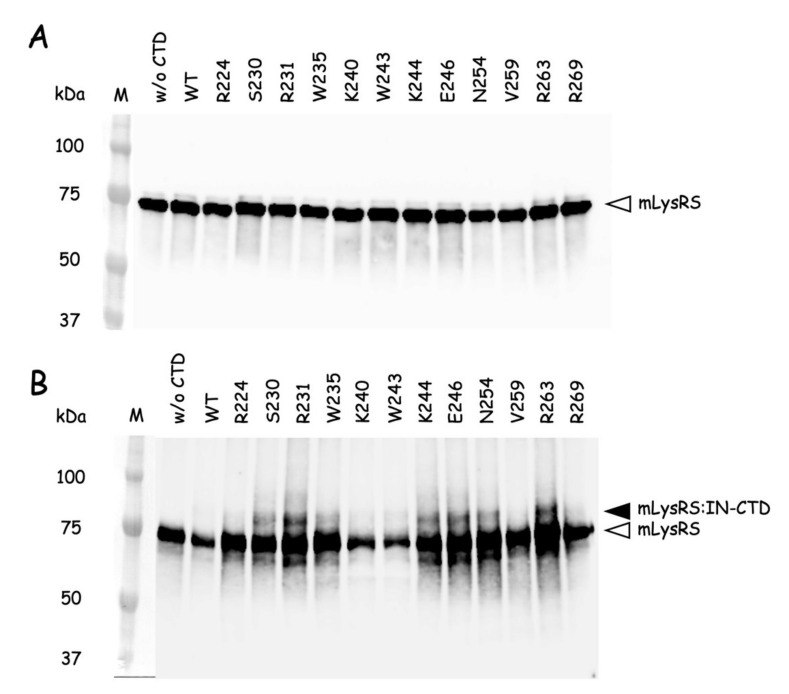
Cross-linking of the *p*Bpa-containing IN-CTD222 variants with mLysRS. Wild-type IN-CTD222 (WT) or IN-CTD222 containing *p*Bpa inserted at different positions (R224 to R269) were incubated at a monomer concentration of 1 µM with mLysRS (dimer concentration of 250 nM) and exposed to UV light at 365 nm for 90 min. Samples recovered before (**A**) or after (**B**) exposure to UV were analyzed by SDS-PAGE and western blotting using anti-LysRS antibodies. The lanes containing the stained size-marker are added on the left (in kDa). The migration level of mLysRS (68 kDa) and of the polypeptide corresponding to an expected cross-linked species containing one monomer of IN-CTD222 and one monomer of mLysRS (79 kDa) are indicated by a white and black arrowhead, respectively. This experiment is the representative of two independent experiments.

To search for residues of the CTD of integrase interacting with residues of mLysRS, 12 mutants of IN-CTD222-H^6^ containing *p*Bpa exposed at the surface of the protein (Table S3) were constructed. The wild-type and *p*Bpa-containing IN-CTD222 proteins were incubated on ice at a monomer concentration of 1.0 µM in the presence of mLysRS at a dimer concentration of 250 nM, in 0.3-fold PBS buffer. After 90 min of exposure to UV at 365 nm, samples were analyzed by SDS-PAGE and western blotting using anti-LysRS antibodies (Figure 6). After incubation with five of the *p*Bpa-containing IN-CTD222 mutants, a high-intensity polypeptide with a molecular mass of 79 kDa was observed, corresponding to a covalent complex containing one monomer of IN-CTD222-H^6^ per monomer of mLysRS. The polypeptide corresponding to the complex was especially visible for IN-CTD222-H^6^ derivatives with *p*Bpa inserted at positions R231 and R263, and to a lesser extent when *p*Bpa was inserted at positions S230, K244, or E246. These five residues are located on one side of the β-barrel structure of IN-CTD222 (Figure S3), which could build the binding interface of integrase for its association with mLysRS.

### 3.5. Molecular Docking of the CTD of Integrase on mLysRS

**Figure 7:**
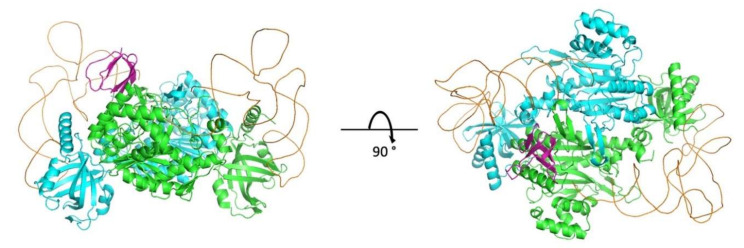
3D model of the mLysRS:IN-CTD complex obtained by molecular docking. The two subunits of LysRS (3BJU) are in cyan and green, the two tRNA molecules in orange, the monomer of IN-CTD (5U1C) in magenta. Only one IN-CTD is shown. The two tRNA molecules were anchored according to the crystal structure of the complex of yeast aspartyl-tRNA synthetase with two tRNA molecules (1ASY). The coordinates of the 3D model can be accessed at https://modelarchive.org/doi/10.5452/ma-bxirn.

In parallel to the identification of the residues involved in the interaction between IN-CTD and mLysRS by experimental methods, we set up an independent docking simulation to explore the most likely interface which could be identified using the InterEvDock2 server [27] and a refinement protocol based on Rosetta software [34] (see Mat & Met). We did not use any of the experimental constraint a priori to guide the docking. Rather, we probed the robustness of the predictive protocol for its ability to retrieve *a posteriori* solutions consistent with experimental data. A good convergence between experimental and computational approaches could then be considered as a proxy for the reliability of the resulting model. At first, 10,000 decoys were sampled by the InterEvDock2 server and a restricted set of 150 most likely rigid-body docked models could be selected. A clustering step narrowed down the number of solutions down to 49 representative models. Despite the challenging size of the complex and the wide heterogeneity of the solutions, a single solution eventually emerged after refinement of the best 49 candidates (Figure 7). The model with the lowest interface energy as estimated by the Rosetta scoring function reached a value significantly lower than any other alternative solution (Figure S4). This feature likely accounts for the significant complementarity of the interactions that could be modeled at the interface of the IN-CTD:mLysRS complex.

This structural model suggests that electrostatic interactions, for instance between LysRS_R228 and IN_E246, or LysRS_E513/514 and IN_R231, as well as hydrophobic packing, for instance between LysRS_F258 and IN_A248, may have a crucial role at the interface between the two proteins (Figure S5). These residues are good candidates to test the validity of the model by mutagenesis. 

### 3.6. Probing the Putative mLysRS:IN-CTD Interface by Site-Directed Mutagenesis and Y2H

The structural model of mLysRS:IN-CTD interaction (Figure 7) suggested a set of mutations that could alter the interaction. Two types of mutations were introduced in mLysRS or in IN-CTD: mutations that are supposed to create electrostatic repulsion between the two proteins, and mutations that have been suggested to alter hydrophobic packing or to introduce steric clashes (Table S4 and Figure S5). Moreover, in some cases, it was envisioned that a mutation in IN-CTD could be compensated by a mutation in mLysRS (for example interaction between residues IN_R263 and LysRS_E531 might be restored in the double mutant IN_R263E and LysRS_E531R). 

**Figure 8:**
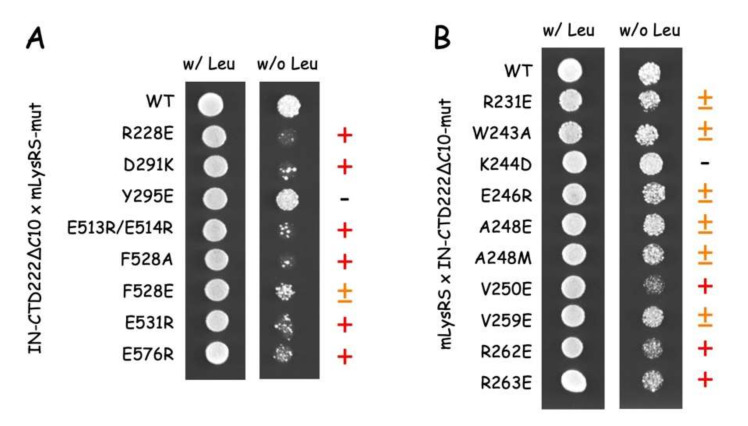
Two-hybrid analysis of mLysRS:IN-CTD interaction. The CTD of HIV-1 integrase (IN-CTD222∆C10) was expressed fused to LexA in pEG202, and mLysRS was expressed fused to the B42 transcription activator under the control of a galactose-inducible promoter in pJG4-5. (**A**) Wild-type IN-CTD222∆C10 was co-expressed with wild-type or mutants of mLysRS. (**B**) Wild-type mLysRS was co-expressed with wild-type or mutants of IN-CTD222∆C10. Yeast cells were grown in a galactose-containing medium in the presence (w/ Leu) or in the absence of leucine (w/o Leu). When the two proteins interact, yeast cells are able to grow in a medium lacking leucine. Mutations have either no effect (-), or have a moderate (±) or strong effect (+) on yeast growth.

The effect of the mutations on the interaction of mLysRS with IN was analyzed in a yeast two-hybrid system previously used to identify the proteins of HIV-1 that interact with mLysRS [8]. Ten mutations were introduced into pEG202 expressing the CTD222∆C10 variant of IN-CTD and eight into pJG4-5 expressing mLysRS (Table S4). Interaction of mLysRS with IN-CTD is reflected by the growth of SKY54 yeast cells on YNBGal medium in the absence of leucine (Figure 8). Mutations R228E, D291K, E513/514R, F528A, E531R, and E576R on mLysRS clearly prevented the growth of SKY54, and mutation F528E had a more moderate effect (Figure 8A). Mutation LysRS_Y295E had no discernible effect. On the other hand, mutations V250E, R262E, and R263E on IN-CTD222∆C10 reduced the growth of SKY54, and mutation R244D had no visible effect on yeast growth (Figure 8B). The six other mutations introduced into the CTD of IN had a more moderate effect. In all cases, when mutations had an effect on yeast growth, no massive-growth was seen, as in the case of wild-type mLysRS and IN-CTD222∆C10, but a punctuated growth was observed indicating that only a fraction of yeast cells expressed the *LEU2* gene and grew, suggesting that an imperfect pair of interacting proteins can activate expression of *LexAop-LEU2*, but at a low frequency.

**Figure 9:**
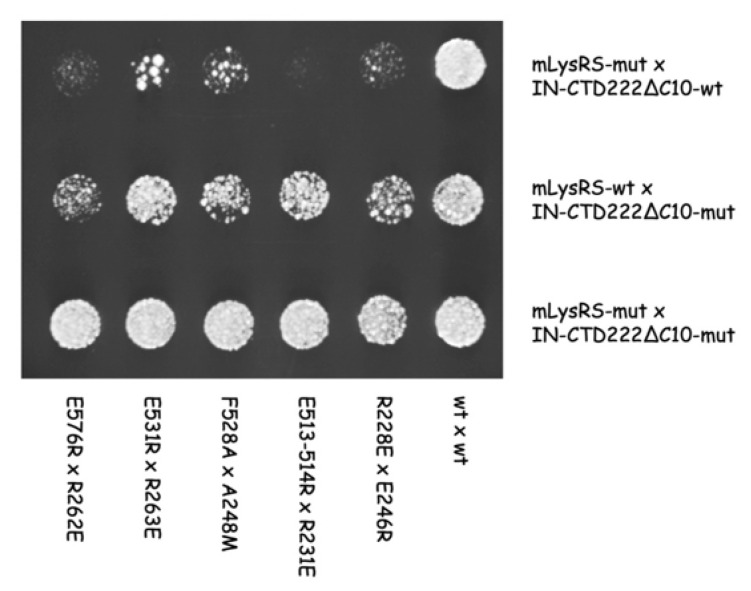
Two-hybrid analysis of compensatory mutations in mLysRS and IN-CTD. Mutants of the CTD of HIV-1 integrase (IN-CTD222∆C10) were co-expressed with mutants of mLysRS in the two-hybrid system, as described in the legend of Figure 8. Cells co-expressing wild-type or mutants of mLysRS with wild-type IN-CTD222∆C10 (first row), wild-type mLysRS with wild-type or mutants of IN-CTD222∆C10 (second row), or a mutant of mLysRS with a mutant of IN-CTD222∆C10 (third row) were grown in a galactose-containing medium in the absence of leucine. Yeast cells expressing compensatory mutations were growing much faster than cells expressing a single of these mutations and were able to grow similarly to cells expressing the wild-type proteins.

Because mLysRS mutations R228E, E513/514R, F528A, E531R, and E576R prevented growth of SKY54 expressing wild-type IN-CTD222∆C10, we anticipated that introduction of a compensatory mutation into IN-CTD222∆C10 might restore growth of SKY54 expressing double mutations (Table S4). Since SKY54 expressing the mutation IN-K244D grew similarly to wild-type (Figure 8), we did not test the combined effect of mutation LysRS_D291K on yeast growth. In all other cases, growth of SKY54 expressing the two mutant proteins was very similar to growth of SKY54 expressing wild-type proteins (Figure 9). This result implies that a permutation of the residues in mLysRS and IN-CTD222∆C10 allowed to re-establish a functional interaction between the two proteins. 

**Figure 10:**
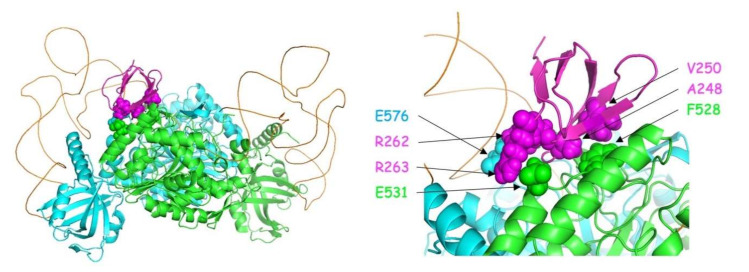
3D model of the platform for association of IN-CTD with mLysRS. **Left**: The structural model shows a dimer of human LysRS in association with a monomer of the CTD of integrase from HIV-1. The two monomers of LysRS (3BJU) are in cyan and green, the two tRNA molecules in orange, the monomer of IN-CTD (5U1C) in magenta. The residues that build the platform for the assembly of the complex are represented by spheres. Docking of the two tRNA molecules was performed using the crystal structure of yeast aspartyl-tRNA synthetase complexed with two tRNA molecules (1ASY). **Right**: Zoom of the IN-CTD:LysRS interaction platform. Residues from the platform are labeled and colored according to the main chains.

The structural model of the mLysRS:IN complex recapitulates the residues of the two partners that have been shown to be crucial for the interaction: A248 and V250 of IN form a patch of hydrophobic residues interacting with F528 from mLysRS; R262 and R263 of IN form salt bridges with E576 and E531 from two monomers of mLysRS, respectively (Figure 10). Insertion of *p*Bpa at positions LysRS_F528, LysRS_E576 and IN_R263 was also found to produce cross-links between the two proteins (Figure 5 and Figure 6). These seven residues build a hydrophobic and electrostatic motif involved in the assembly of the mLysRS:IN complex. Compensatory mutations of residues LysRS_R228 with IN_E246, and LysRS_E513-514 with IN_R231 also restored the interaction (Figure 9). These residues, which are located at the periphery of the interface core, stabilize the interaction. A strong cross-link was also observed when *p*Bpa was inserted at IN-R231 (Figure 6B).

## 4. Discussion

In this work, we obtained a structural model of the complex between mLysRS and IN-CTD strongly supported by three sets of experimental constraints, six cross-links with *p*Bpa-containing mLysRS mutants, five cross-links with *p*Bpa-containing IN-CTD proteins, and five pairs of compensatory mutations. This structural model brings several key insights. First, association of IN-CTD requires that mLysRS is a dimer. Indeed, among the residues clearly involved in the interaction, as assessed by compensatory mutant experiments (Figure 9), two belong to one subunit of the dimer (R228 and E576), and four to the other subunit (E513, E514, F528 and E531) (Figure S5). The two symmetrical binding sites on mLysRS are 30 Å apart, and thus, could bind two IN-CTDs. The polypeptide linkers between IN-CCD and IN-CTD of a dimer of integrase are made of 18 amino acid residues, and were described as non-structured polypeptides in the intasome structure [22] or as long α-helices in the crystal structure of a dimer of IN-CCD-CTD [21]. Therefore, this linker is very flexible and the two CTDs of a dimer of integrase are likely to be able to bind to a dimer of LysRS. This is also consistent with our finding that monomeric IN-CTD binds mLysRS with a *K*_d_ of about 40 to 140 nM (Figure 1C), whereas oligomeric IN binds to a dimer of mLysRS with a 20-fold higher affinity (*K*_d_ of about 3.0 nM, Figure 1A). However, in the context of the GagPol polyprotein, it is not known if a monomeric domain of IN located at the very C-terminus of GagPol interacts with mLysRS to form the GagPol:mLysRS:tRNA^Lys,3^ packaging complex, or if the IN domain of GagPol is able to form a dimer in the context of the polyprotein precursor. The second noticeable feature of the mLysRS-IN complex is the proximity of the tRNA molecules with the CTD domains of integrase (Figure 10). The acceptor stem of the tRNA molecule is close to the IN-CTD, which suggests a possible protein-tRNA interaction that could stabilize the association of the tRNA within the mLysRS:IN complex. In particular, Lys266 and Arg269 are directed towards the TΨC stem-loop of the tRNA molecule and could establish salt bridges. This is consistent with our previous report showing that the affinity of tRNA^Lys,3^ for mLysRS is increased by about two-fold in the presence of a derivative of integrase [12].

The finding that the five compensatory mutants tested in this work (Figure 9) successfully restored normal growth on yeast cells, strongly argue in favor of the proposed model of mLysRS:IN association. It is noteworthy that mutations introduced in IN-CTD have less pronounced growth phenotypes than those introduced in mLysRS (Figure 8). IN-CTD is a small domain made of 57 amino acid residues that form a β-barrel structure (Figure S3). Residues of IN-CTD that build the site of interaction with mLysRS are mostly located in loops that join the β-strands of this small structural domain, which suggests that they may be more mobile than the residues of mLysRS engaged in this assembly platform, which are part of a more compact structure, and are generally located in α-helices. Thus, mutations in IN-CTD could be more easily tolerated due to the flexibility of this domain that could accommodate some variations. 

Among 20,000 sequences of IN from HIV-1 (from non-redundant GenBank CDS translations+PDB+SwissProt+PIR+PRF), only 16 changes are observed for A248 (11S, 4T and 1V), 17 changes for V250 (15I, 2L and 1G), eight changes for R262 (7K and 1G), and 10 changes for R263 (7K, 2G and 1S). This very high level of conservation is also noted among 425 sequences of IN from HIV-2 (from non-redundant GenBank CDS translations+PDB+SwissProt+PIR+PRF), three changes are observed for R262 (1K and 2S), one change for R263 (1G), and A248 is strictly conserved. Concerning V250, this residue is mainly recovered as Ile (390) or Leu (27), corresponding to conservative changes. The high level of conservation of these residues in HIV-1 and HIV-2 suggests that they are important for the function of integrase. Nevertheless, conservation of functionally important residues is likely to be correlated to the many roles of integrase in the life cycle of the virus.

Integrase is a multifunctional protein involved in many aspects of HIV-1 biology. It catalyzes the strand-transfer reaction during the integration step of viral DNA into host genome, it fulfills an essential role in virus morphogenesis, and, as a component of the GagPol polyprotein precursor, it appears to be necessary for the packaging of tRNA^Lys,3^ complexed with mLysRS, a crucial step for initiation of reverse transcription. The CTD of IN is involved in these three functional roles. Residues R228, R231, E246, A248, R263, and K266 are predicted to interact with viral DNA and residues R262, R263, and K266 with another monomer of IN, within the strand-transfer complex [22]. Residues K264, K266, R269, and K273 interact with viral RNA, an essential step in virus morphogenesis [17]. Mutation of these residues into Ala generates noninfectious particles that are unable to initiate reverse transcription. In the present study, mutation of residues R231, W243, E246, A248, V250, V259, R262, and R263 of IN-CTD compromised its interaction with mLysRS and are predicted to abolish tRNA^Lys,3^ packaging into viral particles, and to inhibit viral replication. The conclusions of this work must now be validated by in cellulo approaches. The results obtained in this study offer the opportunity to test several mutants of mLysRS and of the IN domain of GagPol for mLysRS and tRNA^Lys,3^ packaging, for the initiation of reverse transcription of viral RNA and for HIV-1 infectivity.

Although packaging of tRNA^Lys,3^ into new virions is absolutely required to generate infectious particles, several of the conserved residues of IN-CTD identified as key residues in the interaction with mLysRS are also involved in electrostatic protein-DNA interactions within the strand transfer complex [22]. Because residues R231, E246, A248, and R263 are predicted to be involved in these two functions, characterization of the effects of their mutation in a cellular system of HIV-1 replication requires a detailed analysis of the stages of the viral life cycle that are affected by these changes.

Two classes of inhibitors have been developed to inhibit IN functions. INSTIs, such as raltegravir or dolutegravir, inhibit the strand-transfer step catalyzed by IN and are used in antiviral therapies [18]. ALLINIs induce abnormal IN multimerization, prevent interaction on IN with viral RNA, generating eccentric non-infectious particles defective in viral replication [17,19]. Because viral resistance to drugs frequently develops, there is a need to develop antiviral drugs with new resistance profiles. The structural model of the complex between IN-CTD and mLysRS reported in this study will provide a support for searching molecules likely to disrupt the interface between the two proteins. The best inhibitor candidates should be able to interfere with both the hydrophobic and electrostatic components of the assembly platform.

## Supplementary Materials

The following are available online at https://www.mdpi.com/1999-4915/12/10/1202/s1, Figure S1: Characterization of polyclonal anti-IN-CTD antibodies, Figure S2: Localization of *p*Bpa-cross-linked residues on the 3D structure of LysRS, Figure S3: Localization of pBpa-cross-linked residues on the 3D structure of IN-CTD, Figure S4: Pipeline used for the generation of the structural model of mLysRS:IN-CTD complex, Figure S5: Interaction of suggested key residues at the interface of mLysRS with the CTD of HIV-1 integrase, Table S1: Sequence identities between IN species, Table S2: Position of *p*Bpa insertion into mLysRS, Table S3: Position of *p*Bpa insertion into IN-CTD222∆C10 from HIV-1, Table S4: Mutations supposed to alter association of mLysRS with IN-CTD.

## References

[1] Jiang M., Mak J., Ladha A., Cohen E., Klein M., Rovinski B., Kleiman L. (1993). Identification of tRNAs incorporated into wild-type and mutant human immunodeficiency virus type-1. J. Virol..

[2] Pavon-Eternod M., Wei M., Pan T., Kleiman L. (2010). Profiling non-lysyl tRNAs in HIV-1. RNA.

[3] Abbink T.E., Berkhout B. (2007). HIV-1 reverse transcription: Close encounters between the viral genome and a cellular tRNA. Adv. Pharmacol..

[4] Seif E., Niu M., Kleiman L. (2015). In virio SHAPE analysis of tRNA(Lys3) annealing to HIV-1 genomic RNA in wild type and protease-deficient virus. Retrovirology.

[5] Gabor J., Cen S., Javanbakht H., Niu M.J., Kleiman L. (2002). Effect of altering the tRNA_3_^Lys^ concentration in human immunodeficiency virus type 1 upon its annealing to viral RNA, GagPol incorporation, and viral infectivity. J. Virol..

[6] Cen S., Khorchid A., Javanbakht H., Gabor J., Stello T., Shiba K., Musier-Forsyth K., Kleiman L. (2001). Incorporation of lysyl-tRNA synthetase into human immunodeficiency virus type 1. J. Virol..

[7] Kaminska M., Shalak V., Francin M., Mirande M. (2007). Viral hijacking of mitochondrial lysyl-tRNA synthetase. J. Virol..

[8] Kobbi L., Octobre G., Dias J., Comisso M., Mirande M. (2011). Association of mitochondrial lysyl-tRNA synthetase with HIV-1 GagPol involves catalytic domain of the synthetase and transframe and integrase domains of Pol. J. Mol. Biol..

[9] Kovaleski B.J., Kennedy R., Hong M.K., Datta S.A., Kleiman L., Rein A., Musier-Forsyth K. (2006). In vitro characterization of the interaction between HIV-1 Gag and human lysyl-tRNA synthetase. J. Biol. Chem..

[10] Duchon A.A., St Gelais C., Titkemeier N., Hatterschide J., Wu L., Musier-Forsyth K. (2017). HIV-1 exploits a dynamic multi-aminoacyl-tRNA synthetase complex to enhance viral replication. J. Virol..

[11] Dias J., Octobre G., Kobbi L., Comisso M., Flisiak S., Mirande M. (2012). Activation of human mitochondrial lysyl-tRNA synthetase upon maturation of its premitochondrial precursor. Biochemistry.

[12] Khoder-Agha F., Dias J., Comisso M., Mirande M. (2018). Characterization of association of human mitochondrial lysyl-tRNA synthetase with HIV-Pol and tRNA(Lys,3). BMC Biochem..

[13] Tolkunova E., Park H., Xia J., King M.P., Davidson E. (2000). The human lysyl-tRNA synthetase gene encodes both the cytoplasmic and mitochondrial enzymes by means of an unusual alternative splicing of the primary transcript. J. Biol. Chem..

[14] Francin M., Kaminska M., Kerjan P., Mirande M. (2002). The N-terminal domain of mammalian lysyl-tRNA synthetase is a functional tRNA-binding domain. J. Biol. Chem..

[15] Guo M., Ignatov M., Musier-Forsyth K., Schimmel P., Yang X.L. (2008). Crystal structure of tetrameric form of human lysyl-tRNA synthetase: Implications for multisynthetase complex formation. Proc. Natl. Acad. Sci. USA.

[16] Deprez E., Tauc P., Leh H., Mouscadet J.F., Auclair C., Brochon J.C. (2000). Oligomeric states of the HIV-1 integrase as measured by time-resolved fluorescence anisotropy. Biochemistry.

[17] Kessl J.J., Kutluay S.B., Townsend D., Rebensburg S., Slaughter A., Larue R.C., Shkriabai N., Bakouche N., Fuchs J.R., Bieniasz P.D. (2016). HIV-1 integrase binds the viral RNA genome and is essential during virion morphogenesis. Cell.

[18] Brooks K.M., Sherman E.M., Egelund E.F., Brotherton A., Durham S., Badowski M.E., Cluck D.B. (2019). Integrase inhibitors: After 10 years of experience, is the best yet to come?. Pharmacotherapy.

[19] Koneru P.C., Francis A.C., Deng N., Rebensburg S.V., Hoyte A.C., Lindenberger J., Adu-Ampratwum D., Larue R.C., Wempe M.F., Engelman A.N. (2019). HIV-1 integrase tetramers are the antiviral target of pyridine-based allosteric integrase inhibitors. eLife.

[20] Wang J.-Y., Ling H., Yang W., Craigie R. (2001). Structure of a two-domain fragment of HIV-1 integrase: Implications for domain organization in the intact protein. EMBO J..

[21] Chen J.C.-H., Krucinski J., Miercke L.J.W., Finer-Moore J.S., Tang A.H., Leavitt A.D., Stroud R.M. (2000). Crystal structure of the HIV-1 integrase catalytic core and C-terminal domains: A model for viral DNA binding. Proc. Natl. Acad. Sci. USA.

[22] Passos D.O., Li M., Yang R., Rebensburg S.V., Ghirlando R., Jeon Y., Shkriabai N., Kvaratskhelia M., Craigie R., Lyumkis D. (2017). Cryo-EM structures and atomic model of the HIV-1 strand transfer complex intasome. Science.

[23] Li M.Z., Elledge S.J. (2007). Harnessing homologous recombination in vitro to generate recombinant DNA via SLIC. Nat. Methods.

[24] Rémion A., Khoder-Agha F., Cornu D., Argentini M., Redeker V., Mirande M. (2016). Identification of protein interfaces within the multi-aminoacyl-tRNA synthetase complex: The case of lysyl-tRNA synthetase and the scaffold protein p38. FEBS Open Bio.

[25] Ryu Y., Schultz P.G. (2006). Efficient incorporation of unnatural amino acids into proteins in Escherichia coli. Nat. Methods.

[26] Farrell I.S., Toroney R., Hazen J.L., Mehl R.A., Chin J.W. (2005). Photo-cross-linking interacting proteins with a genetically encoded benzophenone. Nat. Methods.

[27] Quignot C., Rey J., Yu J., Tuffery P., Guerois R., Andreani J. (2018). InterEvDock2: An expanded server for protein docking using evolutionary and biological information from homology models and multimeric inputs. Nucleic Acids Res..

[28] Ramirez-Aportela E., Lopez-Blanco J.R., Chacon P. (2016). FRODOCK 2.0: Fast protein-protein docking server. Bioinformatics.

[29] Dong G.Q., Fan H., Schneidman-Duhovny D., Webb B., Sali A. (2013). Optimized atomic statistical potentials: Assessment of protein interfaces and loops. Bioinformatics.

[30] Andreani J., Faure G., Guerois R. (2013). InterEvScore: A novel coarse-grained interface scoring function using a multi-body statistical potential coupled to evolution. Bioinformatics.

[31] Berto A., Yu J., Morchoisne-Bolhy S., Bertipaglia C., Vallee R., Dumont J., Ochsenbein F., Guerois R., Doye V. (2018). Disentangling the molecular determinants for Cenp-F localization to nuclear pores and kinetochores. EMBO Rep..

[32] Nadaradjane A.A., Quignot C., Traore S., Andreani J., Guerois R. (2020). Docking proteins and peptides under evolutionary constraints in Critical Assessment of PRediction of Interactions rounds 38 to 45. Proteins.

[33] Rodrigues J.P., Trellet M., Schmitz C., Kastritis P., Karaca E., Melquiond A.S., Bonvin A.M. (2012). Clustering biomolecular complexes by residue contacts similarity. Proteins.

[34] Leman J.K., Weitzner B.D., Lewis S.M., Adolf-Bryfogle J., Alam N., Alford R.F., Aprahamian M., Baker D., Barlow K.A., Barth P. (2020). Macromolecular modeling and design in Rosetta: Recent methods and frameworks. Nat. Methods.

[35] Gyuris J., Golemis E., Chertkov H., Brent R. (1993). Cdi1, a human G1 and S phase protein phosphatase that associates with Cdk2. Cell.

[36] Khazak V., Kato-Stankiewicz J., Tamanoi F., Golemis E.A. (2006). Yeast screens for inhibitors of Ras-Raf interaction and characterization of MCP inhibitors of Ras-Raf interaction. Methods Enzym..

[37] Mirande M., Cirakoglu B., Waller J.P. (1982). Macromolecular complexes from sheep and rabbit containing seven aminoacyl-tRNA synthetases. III. Assignment of aminoacyl-tRNA synthetase activities to the polypeptide components of the complexes. J. Biol. Chem..

