# Supplementary material for: How HIV-1 Integrase Associates with Human Mitochondrial Lysyl-tRNA Synthetase
Source: Viruses. 2020 Oct 21;12(10):1202. doi: 10.3390/v12101202 (PMC7589778; doi:10.3390/v12101202)
Supplement: Supplementary file 1 [file viruses-12-01202-s001.pdf]

Supplementary Materials

How HIV-1 integrase associates with human mitochondrial lysyl-tRNA synthetase ?

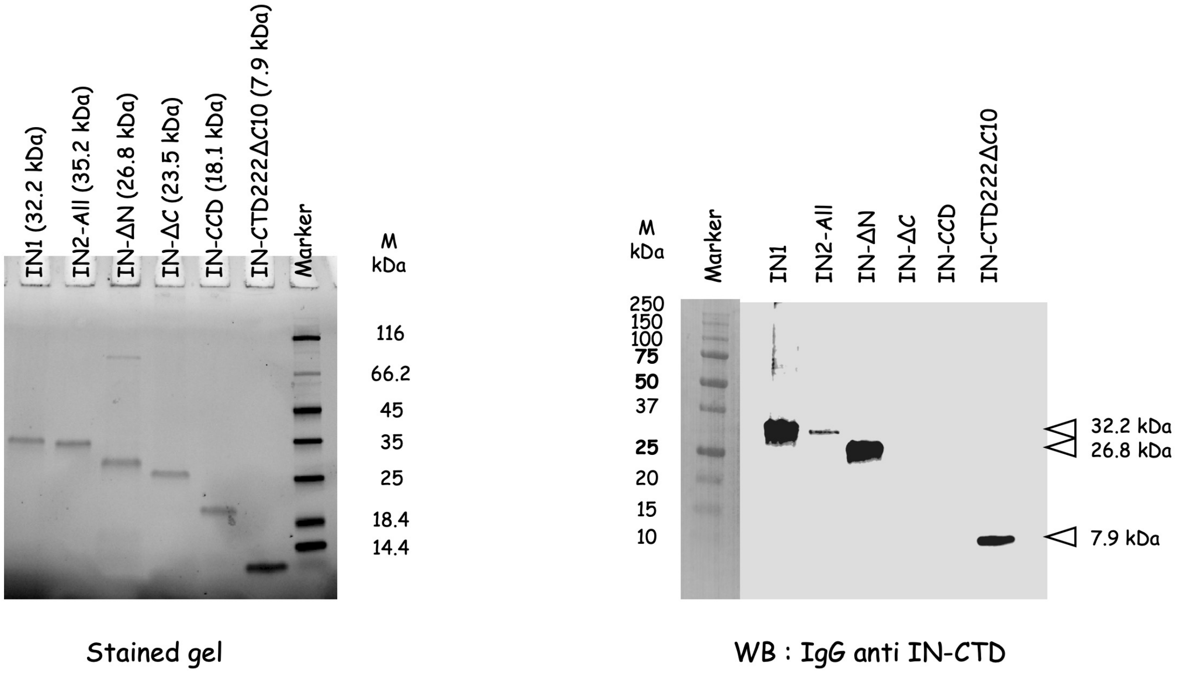

Figure S1: Characterization of polyclonal anti-IN-CTD antibodies. Different constructs of integrase (100 ng of protein) were separated by SDS-PAGE on a stain-free gel (Biorad) (left) and subjected to Western blotting using a polyclonal antibody (GeneCust) raised against the CTD of IN from HIV-1 (right). The different IN species are : IN-HIV-1 (IN1), IN-HIV-2_ALL (IN2-All), IN-HIV-1 with a deletion of the NTD (IN-ΔN), IN-HIV-1 with a deletion of the CTD (IN-ΔC), the CCD of IN-HIV-1 (IN-CCD), the CTD222ΔC10 domain of IN-HIV-1 (IN-CTD222ΔC10).

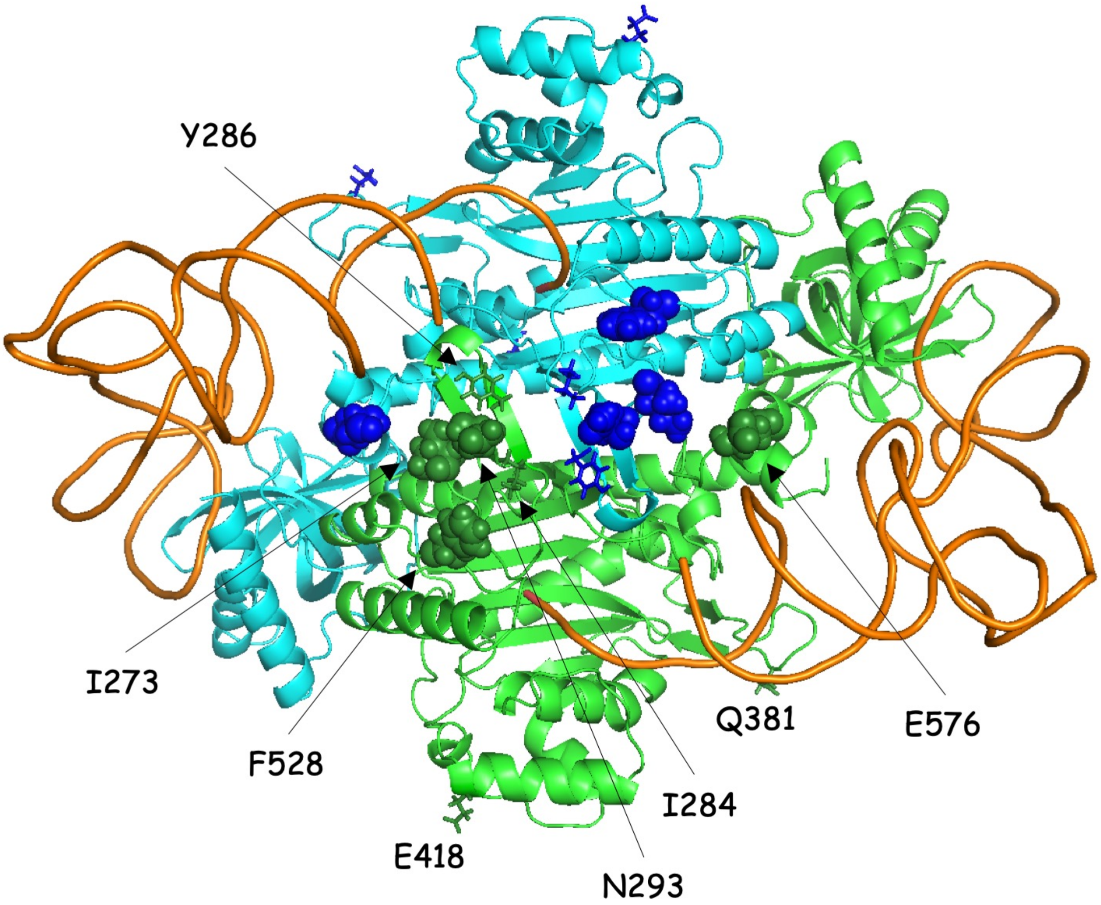

Figure S2: Localization of *p*Bpa-cross-linked residues on the 3D-structure of LysRS. The two monomers are in green and cyan, the two tRNA molecules are in orange. Side-chains of residues I273, N293, F528 and E576 are indicated by spheres, and of residues I284, Y286, H364, Q381 and E418 are indicated by sticks.

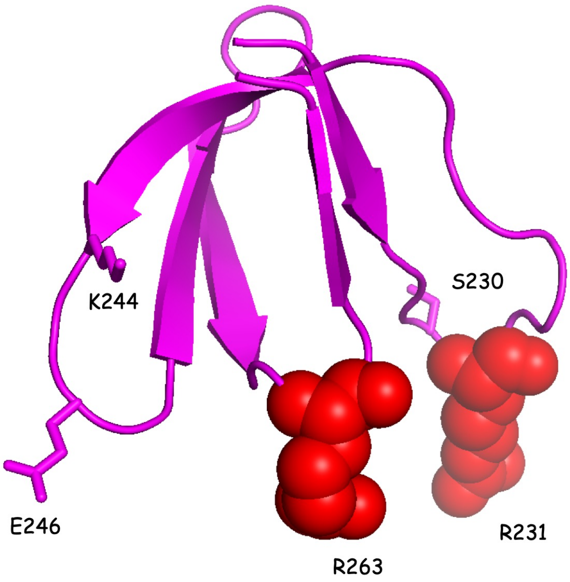

Figure S3: Localization of pBpa-cross-linked residues on the 3D-structure of IN-CTD. Side-chains of residues R231 and R263 are indicated by spheres in red, and of residues S230, K244 and E246 are indicated by spheres in magenta.

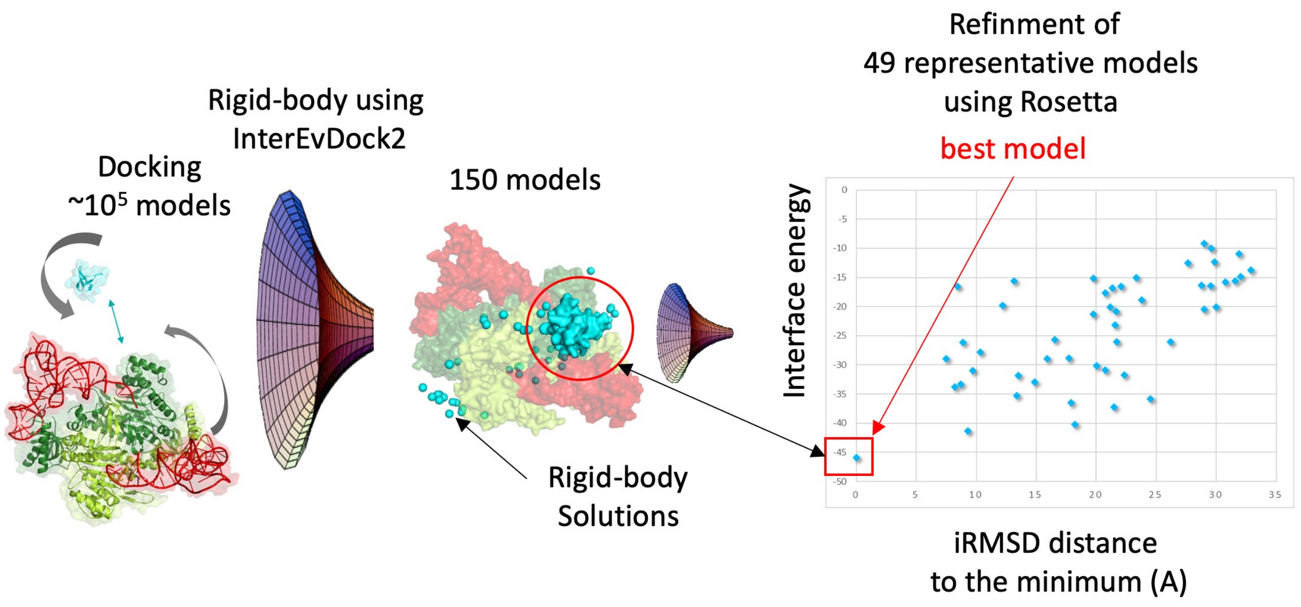

Figure S4: Pipeline used for the generation of the structural model of mLysRS:IN-CTD complex. InterEvDock2 server including the frodock rigid-body algorithm was used to generate 10^5 decoys, rescore the best 10^4 decoys by a consensus score and extract the best 150 decoys fulfilling InterEvDock2 consensus. After clustering, a subset of 49 representative models were further refined using Rosetta. The centroïds of the 150 IN-CTD domains are represented as cyan dots docked against the green surface representation of mLysRS dimer with tRNA shown in red. The model of IN-CTD domain having the best interface energy after refinement is shown in cyan surface. The interface energy of the best model is plotted on the right panel versus the interface RMSD between that model and the 48 alternative refined models. The model with best interface energy is highly consistent with most of the experimental constraints generated in the study and its coordinates can be accessed at https://modelarchive.org/doi/10.5452/ma-bxirn .

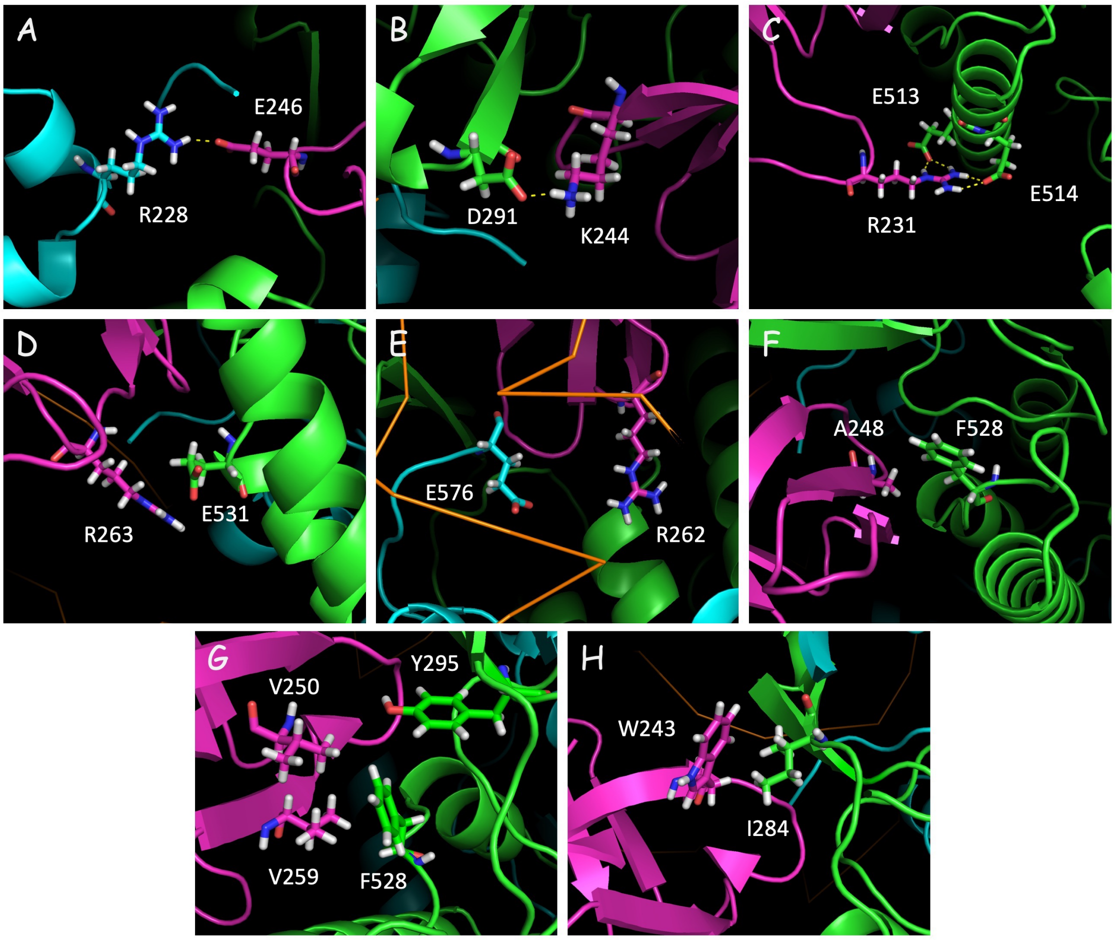

Figure S5: Interaction of suggested key residues at the interface of mLysRS with the CTD of HIV-1 integrase. The two monomers of mLysRS are shown in cyan and green, IN-CTD is shown in magenta. The residues suggested to play an important role in the interaction are depicted as sticks. The backbone of the tRNA molecule is shown in orange. The possible salt bridges are depicted as yellow dashed lines. (A) IN_E246 and LysRS_R228; (B) IN_K244 and LysRS_D291; (C) IN_R231 and LysRS_E513/514; (D) IN_R263 and LysRS_E531; (E) IN_R262 and LysRS_E576; (F) IN_A248 and LysRS_F528; (G) IN_V250, IN_V259 and LysRS_Y295, LysRS_F528; (H) IN_W243 and LysRS_I284.

Table S1: Sequence identities between IN species

Domain	Species 1 / Species 2		Identities (%)
IN	HIV-1	HIV-2_TRA	60.1
	HIV-1	HIV-2_ALL	59.7
	HIV-2_TRA	HIV-2_ALL	96.5
IN-NTD	HIV-1	HIV-2_TRA	53.8
	HIV-1	HIV-2_ALL	53.8
	HIV-2_TRA	HIV-2_ALL	97.4
IN-CCD	HIV-1	HIV-2_TRA	64.1
	HIV-1	HIV-2_ALL	64.1
	HIV-2_TRA	HIV-2_ALL	96.6
IN-CTD	HIV-1	HIV-2_TRA	71.1
	HIV-1	HIV-2_ALL	68.9
	HIV-2_TRA	HIV-2_ALL	97.8

Table S2: Position of *p*Bpa insertion into mLysRS

Residue	Position in LysRS+	Position in pmLysRS*
Asp	222	250
Phe	239	267
Glu	260	288
Glu	267	295
Ile	273	301
Ile	284	312
Tyr	286	314
Asn	288	316
Asp	291	319
Asn	293	321
Tyr	295	323
Lys	356	384
His	364	392
Lys	370	398
Glu	379	407
Gln	381	409
Asp	384	412
Arg	392	420
Glu	398	426
Lys	402	430
Met	406	434
Glu	410	438
Glu	418	446
Lys	421	449
Val	428	456
Pro	436	464
Arg	477	505
Gln	510	538
Lys	517	545
Ala	520	548
Asp	524	552
Phe	528	556
Glu	531	559
Phe	570	598
Glu	576	604

+ numbering is according to PDB file 3BJU for cytoplasmic LysRS
* numbering is according to the sequence of premitochondrial LysRS encoded in AF285758.

Table S3: Position of *p*Bpa insertion into IN-CTD222ΔC10

Residue	Position in IN-CTD+
Arg	224
Ser	230
Arg	231
Trp	235
Lys	240
Trp	243
Lys	244
Glu	246
Asn	254
Val	259
Arg	263
Arg	269

+ numbering is according to PDB file 5U1C

Table S4: Mutations supposed to alter association of mLysRS with IN-CTD **

Mutations in mLysRS	Mutations in IN-CTD
Mutations supposed to create electrostatic repulsion	
LysRS_R228E	IN_E246R
LysRS_D291K	IN_K244D
LysRS_E513/514R *	IN_R231E
LysRS_E531R	IN_R263E
LysRS_E576R	IN_R262E
Mutations supposed to alter hydrophobic interactions	
LysRS_Y295E	-
LysRS_F528A	IN_A248M
LysRS_F528E	-
-	IN_W243A
-	IN_A248E
-	IN_V250E
-	IN_V259E

*double mutant E513R and E514R

**mutations in mLysRS that are supposed to be compensated by a mutation in IN-CTD are listed side by side
